# 497. Safety and tolerability of 2000mg intravenous sotrovimab dose in immunocompromised participants uninfected with SARS-CoV-2 in the PROTECT-V trial

**DOI:** 10.1093/ofid/ofad500.566

**Published:** 2023-11-27

**Authors:** Davinder Dosanjh, Wendi Qian, Francis Dowling, Louise Crowley, Michael Chen-Xu, Rona M Smith

**Affiliations:** University of Birmingham, Birmingham, Northern Ireland, United Kingdom; Cambridge University Hospitals NHS Foundation Trust, Cambridge, England, United Kingdom; Cambridge University Hospitals NHS Foundation Trust, Cambridge, England, United Kingdom; University of Birmingham, Birmingham, Northern Ireland, United Kingdom; University of Cambridge, Cambridge, England, United Kingdom; University of Cambridge, Cambridge, England, United Kingdom

## Abstract

**Background:**

There remains a need for pre-exposure prophylaxis against SARS-CoV-2 infection in vulnerable patients in whom response to vaccination is often sub-optimal. The PROTECT-V platform trial is testing pre-exposure prophylactic interventions for COVID-19 in vulnerable patient populations: transplant recipients, individuals with oncological/haematological diagnoses, immune deficiency, autoimmune diseases requiring immunosuppression, and individuals receiving dialysis.

**Methods:**

Sotrovimab is a dual-action monoclonal antibody and the second agent to be added to the PROTECT-V platform (Clinicaltrials.gov: NCT04870333; EudraCT: 2020-004144-28). Although a single sotrovimab 500mg intravenous (IV) dose has been widely used for early treatment, data on a 2000mg IV dose are limited. Tolerability data in the first 143 participants randomized to this arm of the trial are presently available. Patients are randomized 1:1 sotrovimab to placebo. Data remain blinded.

**Results:**

Median age was 66 years (range 21 – 86) and 82 (57%) patients were female. 132 (96%) had received ≥3 doses of SARS-CoV-2 vaccine. Patient populations were: 82 (57%) autoimmune disease; 26 (18%) haematological/oncological diseases; 21 (15%) transplant recipients; 12 (8%) immunodeficiency; 2 (1%) on dialysis.

Two (1%) participants experienced a mild infusion related reaction (IRR). The infusion was briefly interrupted, but completed. Thirty (21%) participants experienced at least one symptom in the 24 hours post-infusion, but none were severe or required hospital admission. The most common symptoms were dizziness (7 [5%]), headache (7 [5%]), rigors (5 [4%]) and fever (4 [3%]). No severe adverse events were reported within 72 hours of IMP infusion at the time of writing.

The first 55 patients underwent routine hematological and biochemical blood test evaluation 72 hours post-infusion. Ten events from 9 participants exhibited worsening of laboratory parameters, meeting at least grade two DAIDS criteria or worse. None of these were clinically significant.

**Conclusion:**

A 2000mg IV dose of sotrovimab was tolerated well in this blinded analysis of immunocompromised participants, with no severe IRRs or significant change in haematological or biochemical markers up to 72 hours post infusion.

**Disclosures:**

**Davinder Dosanjh, n/a**, Astrazeneca: Honoraria|Astrazeneca: Employee|Boehringer Ingelheim: Advisor/Consultant|Boehringer Ingelheim: Honoraria|Gilead: Advisor/Consultant|GSK: Grant/Research Support|Synairgen: Advisor/Consultant **Louise Crowley, n/a**, GSK: Grant/Research Support **Michael Chen-Xu, n/a**, GSK: Grant/Research Support **Rona M. Smith, MD MRCP**, GSK: Grant/Research Support|Union Therapeutics: Grant/Research Support

